# Emergency Physicians: Beware of the Consent Standard of Care

**DOI:** 10.5811/cpcem.2018.1.37822

**Published:** 2018-04-05

**Authors:** Gregory P. Moore, Aaron G. Matlock, John L. Kiley, Katherine D. Percy

**Affiliations:** * Madigan Army Medical Center, Department of Emergency Medicine, Tacoma, Washington; † Brooke Army Medical Center, Department of Emergency Medicine, San Antonio, Texas; ‡ Brooke Army Medical Center, Department of Internal Medicine, San Antonio, Texas; § Tripler Army Medical Center, Department of Emergency Medicine, Honolulu, Hawaii

## Abstract

Many emergency physicians view informed consent as a necessary component of treatments or procedures to be performed on their patients. When such procedures are necessary, often there is a discussion of risks, benefits and alternatives with forms signed to validate the discussion. Two Wisconsin emergency department medical-legal cases have expanded liability of the duty of informed consent. These cases have focused on withholding medication and diagnostic tests.

## INTRODUCTION

We present two Wisconsin cases that illustrate a frequent trend of litigation in medical malpractice across the nation. When a bad medical outcome occurs, plaintiff attorneys will bring suit for both medical malpractice and lack of informed consent. The informed consent malpractice will allege that the patient was not given information about available tests, treatments, or admission to the hospital. This gives the plaintiffs two lines of attack and possible recovery. It is not enough in all cases to provide the standard of care medically.

### CASE 1: *Jandre v Physicians Insurance Co of Wisconsin*

Mr. Thomas Jandre, a 48-year-old male, was brought to a Wisconsin emergency department (ED) by his co-workers. While traveling to a jobsite, coffee he was drinking had come out of his nose, and he developed slurred speech, left-sided facial droop, dizziness and unsteadiness with leg weakness. On presentation, the findings were noted by co-workers and an ED nurse. The patient was then evaluated by the emergency physician (EP). After the history and physical (which included auscultation of the carotid arteries for bruits), a computed tomography of the patient’s head was obtained and interpreted by a radiologist as normal. The EP elected not to obtain a carotid artery ultrasound and diagnosed Mr. Jandre with Bell’s palsy. The EP prescribed medications and discharged the patient with instructions to see a neurologist for follow-up care.

Three days later, a family physician agreed that Mr. Jandre had Bell’s palsy that was resolving. Eleven days after the initial ED visit, Mr. Jandre suffered an ischemic stroke that left him physically and cognitively impaired. A carotid ultrasound revealed a 95% occlusion of the right internal carotid artery. The Jandres filed a lawsuit alleging two offenses. The first was that the EP negligently diagnosed Jandre with Bell’s palsy, and the second was that the EP “breached her duty to inform” the patient of an additional test (a carotid ultrasound) that was available to assist in the evaluation of a potential ischemic stroke.

With regard to the first offense, the jury was educated about the “reasonable doctor” standard of care, in which the physician can be found negligent if they “failed to use the degree of care, skill, and judgment which reasonable emergency room physicians would exercise given the state of medical knowledge.” There was evidence that the EP used acceptable methods for diagnosis and treatment, which the jury considered reasonable, and the EP was found to be non-negligent with respect to misdiagnosis. There was no contention of this verdict by the Jandres.

The second offense alleged that the EP was negligent with respect to her duty to inform the patient of the possibility of an additional diagnostic procedure. This brought into question the “reasonable patient standard,” which states that “a doctor must provide the patient with the information a reasonable person in the patient’s position would regard as significant when deciding to accept or reject a diagnostic procedure.” The patient’s family felt that the EP had not told them that the patient had an atypical presentation of Bell’s palsy, that the symptoms could also be consistent with an acute ischemic stroke, that she had used a less-reliable method to rule out carotid pathology (auscultation rather than ultrasound).

In both the original jury trial and at appeal, it was decided that the physician was not negligent in her medical care, but was separately negligent in her duty to disclose. The co-defendant (Physicians Insurance Co. of Wisconsin) independently appealed the case to the Supreme Court of Wisconsin with the following question: “Is there a bright-line rule that once a physician makes a non-negligent final diagnosis, there is no duty to inform the patient about diagnostic tests for conditions unrelated to the condition that was included in the final diagnosis?” The defendants argued that a reasonable patient would not need information regarding tests and treatment options unrelated to the diagnosed condition. They noted inconsistencies between the first ruling and the second allegation and argued that they could not be held liable for a duty to inform when the physician was not found negligent in her care or diagnosis. Additionally, the defendants argued that they were supported by two prior court rulings that stated duty to inform applies to treatment, not diagnostic tests. They contended that the duty to inform only applied after reaching a final diagnosis and not during the diagnostic process.

In response, the Jandres argued that a reasonable patient would have wanted to know if an additional non-invasive imaging modality could more accurately assess the patient’s carotid arteries. Additionally, they argued that a jury can apply different standards of care for each offense without them being contradictory. The court remanded the case back to a jury decision solely on the lack of informed consent offense. The jury decided that the EP was negligent with respect to duty to inform and awarded damages of approximately $1.853 million.^1^

### CASE 2: *Mayo v Jaffe*

Ascaris Mayo, a 53-year-old female, went to the ED of Columbia St. Mary’s Hospital in Milwaukee complaining of abdominal pain and a high fever. She was seen by the EP and a physician’s assistant; at trial they admitted that they had “included infection in the differential diagnosis” and that Mayo “met the criteria for Systematic Inflammatory Response Syndrome,” according to court records. Ms. Mayo wasn’t told about the diagnosis or available treatment, such as antibiotics. Instead, she was told to consult with her gynecologist about her history of uterine fibroids, court records show. Her condition worsened and she went to another ED the next day, where she was diagnosed with Group A sepsis. Mayo developed multi-organ system failure and went into a coma.

While in the hospital all of her extremities became gangrenous and required amputation. Mayo and her husband sued both providers along with Infinity Health Care Inc., ProAssurance Wisconsin Insurance Company and the Wisconsin Injured Patients and Families Compensation Fund, for medical malpractice and failure to provide proper informed consent. The jury found that neither provider was negligent but that both failed to provide Mayo with proper informed consent about her diagnosis and treatment choices. The jury awarded $25.3 million.^2^

## DISCUSSION

### Dr. Percy

The 1972 landmark case of Canterbury v Spence defined informed consent and established *failure to inform* as a distinct area of medical negligence, separate from actual care provided. *Canterbury v Spence* stated that it is the duty of the physician to disclose all information that a reasonable person would deem important to make an informed decision irrespective of whether they would comply with the care suggested for them. This court established that informed consent requires a discussion including inherent and potential hazards, benefits, and alternatives that a reasonable person of average intelligence would want before making a decision about their care. Also required is discussion of risk vs. benefit, expected outcome without care, and incidence of injury, harm, death and disability, along with all information that if provided could change the patient’s mind about proceeding with care.^3^

### Dr. Moore

When juries and courts have a lawsuit, there are two possible standards that can be applied: A) “reasonable patient” standard of care or, B) “reasonable physician” standard of care. At the time of these trials in Wisconsin, the patient-centered standard of care was in effect and the law for consent was “what a reasonable person in the patient’s position would want to know.”^4^ Due to outcry from these verdicts, the Wisconsin law was changed to the “reasonable physician standard.” The new law “requires disclosure only of information that a reasonable physician in the same or similar medical specialty would know and disclose under the circumstances.”^5^ Each state adopts one or the other, or in some cases their own unique standard. This is accomplished by either legislation, or individual case decision in the state courts. About half of the states follow the patient-centered standard and about half follow the physician-centered standard.^6^ It is harder for a plaintiff to succeed in a lawsuit successfully if the physician standard is in effect.

### Dr. Matlock

It is evident that in many medical-legal cases, there can be a claim for both negligence and lack of informed consent. These are distinct and separate issues. The elements that must be proven to successfully litigate a claim of lack of informed consent are 1), “the physician did not present the risks and benefits of the proposed treatment and or alternative treatments; 2) with full information, the patient would have declined the treatment; and 3) the treatment, even though appropriate and carried out skillfully, was a substantial factor causing the patient’s injuries.”^7^,^8^

### Dr. Kiley

These types of cases are not isolated to Wisconsin. This legal argument has been made recently in a Louisiana trial case as well. In *Coulon v Creel*, a patient with a stroke, not treated with tissue plasminogen activator (tPA), sued in a similar double-pronged argument. They claimed both medical malpractice and secondly for malpractice with regard to informed consent. At trial the jury awarded Mr. Coulon $150,000 in damages, specifically noting the lack of documented discussion of tPA being withheld. It seems evident that the jury, when determining the relatively small size of the award, was not convinced that tPA should have been definitely administered. They were unwilling to provide for future medical care. However, the jury seemed willing to provide some compensation for unequivocal lack of documentation of a disputed informed disclosure. The amount of the award was appealed. A higher court upheld the jury award and did not allow for increased damages.^9^,^10^

## CONCLUSION

Review of the defining medical-legal cases and precedents may both confuse and frustrate well-intentioned physicians who aspire to have clear guidelines and precisely defined duties. With regard to informed consent, the duty to inform can appear cloudy and nebulous. The cases reviewed here reveal that when a provider is in doubt as to whether to disclose information, it may be optimal to err on the side of discussion rather than to withhold information. While this may be cumbersome, in general, courts have not allowed time constraints as sufficient to excuse this duty. It is also important to clearly document that information has been shared and discussed (as illustrated by the Coulon case above).

### Take-home Points

1. The duty to disclose (informed consent) of an EP extends to areas beyond procedures and includes patient disposition, and test ordering.
2. It behooves EPs to have open discussions and disclosure in situations where a reasonable patient would desire more information and consider this shared decision-making in critical test-ordering and dispositions.
3. Failure to do so may increase liability.

Shared decision-making /informed consent discussions should be documented to increase medical-legal protection.

Documented patient informed consent and/or Institutional Review Board approval has been obtained and filed for publication of this case report.
